# Intraoperative use of extracorporeal CO_2_ removal (ECCO_2_R) and emergency ECMO requirement in patients undergoing lung transplant: a case-matched cohort retrospective study

**DOI:** 10.1186/s44158-022-00050-x

**Published:** 2022-05-24

**Authors:** Franco Ruberto, Francesco Alessandri, Mario Piazzolla, Veronica Zullino, Katia Bruno, Paola Celli, Daniele Diso, Federico Venuta, Federico Bilotta, Francesco Pugliese

**Affiliations:** Department of General and Specialistic Surgery “Paride Stefanini”, “Sapienza” University of Rome, Viale del Policlinico 155, 00161 Rome, Italy

**Keywords:** Lung transplant, ECCO2R, Hypercapnia, Intraoperative ECLS

## Abstract

**Background:**

The use of extracorporeal carbon dioxide removal (ECCO_2_R) is less invasive than extracorporeal membrane oxygenation (ECMO), and intraoperative control of gas exchange could be feasible. The aim of this study in intermediate intraoperative severity patients undergoing LT was to assess the role of intraoperative ECCO_2_R on emergency ECMO requirement in patients.

**Methods:**

Thirty-eight consecutive patients undergoing lung transplantation (LT) with “intermediate” intraoperative severity in the intervals 2007 to 2010 or 2011 to 2014 were analyzed as historical comparison of case-matched cohort retrospective study. The “intermediate” intraoperative severity was defined as the development of intraoperative severe respiratory acidosis with maintained oxygenation function (i.e., pH <7.25, PaCO_2_ >60 mmHg, and PaO_2_/FiO_2_ >150), not associated with hemodynamic instability. Of these 38 patients, twenty-three patients were treated in the 2007–2010 interval by receiving “standard intraoperative treatment,” while 15 patients were treated in the 2011–2014 interval by receiving “standard intraoperative treatment + ECCO_2_R.”

**Results:**

ECMO requirement was more frequent among patients that received “standard intraoperative treatment” alone than in those treated with “standard intraoperative treatment + ECCO_2_R” (17/23 vs. 3/15; *p* = 0.004). The use of ECCO_2_R improved pH and PaCO_2_ while mean pulmonary artery pressure (mPAP) decreased.

**Conclusion:**

In intermediate intraoperative severity patients, the use of ECCO_2_R reduces the ECMO requirement.

## Background

Perioperative management of patients undergoing lung transplant (LT) encompass to address serious respiratory and cardiac complications including systemic hypotension, pulmonary hypertension, right ventricular dysfunction, hypercapnia, and hypoxemia [1, 2]. Although LT is generally performed off-pump, extracorporeal support is necessary for up to 30 to 40% of the patients in whom various cardiopulmonary assistance techniques have been used [2–4]. The use of cardiopulmonary bypass (CPB), introduced in clinical practice since in the early 1980s, radically changed the approach to LT in more severely sick patients and allowed to treat cases in extremely serious conditions [5]. On the other hand, CPB implies the use of anticoagulants and the related increase in bleeding and inflammation associated with primary graft dysfunction (PGD) and pulmonary complications [6, 7]. Perioperative use of extracorporeal membrane oxygenation (ECMO) has been subsequently introduced in LT for rescue treatment of severe respiratory failure and advanced hemodynamic instability [6, 7]. Due to the centrifugal pump and closed-circuit membrane oxygenator, ECMO overcomes some of the CPB-related limitations as the necessity for high-dose heparin, the wide air-blood interface, and systemic inflammatory reaction [7, 8].

Extracorporeal carbon dioxide removal (ECCO_2_R) has been successfully used in moderate ARDS to selectively reduce arterial carbon dioxide partial pressure (PaCO_2_) [9]. This technique has the potential advantage, when compared to CPB and ECMO, of being partially associated with lower anticoagulation and device-related complications, with less invasiveness (i.e., single venous double-lumen catheter) [10]. Despite ECCO_2_R use is not associated with improved oxygenation, this device induces PaCO_2_ reduction by a single venous double lumen catheter at a lower flow rate than ECMO [11, 12]. In thoracic surgery, ECCO_2_R has been used before LT as a “bridge” to organ availability and in patients with PGD after LT, but there is no report on intraoperative ECCO_2_R use during LT [13, 14].

This case-matched cohort retrospective study in patients undergoing LT is intended to evaluate the role of intraoperative ECCO_2_R on emergency ECMO requirements.

## Methods

The clinical records of all consecutive patients undergoing LT at Transplant Centre of Policlinico Umberto I, University of Rome “Sapienza”, Rome, Italy, between November 2007 and March 2014, were retrieved with the approval of the Ethical Committee of Policlinico Umberto I (Protocol Number 756/13). Patients were categorized according to the clinical conditions into 3 groups: “high”, “low,” and “intermediate” intraoperative severity. Patients receiving preoperatively extracorporeal lung support (ECMO or ECCO_2_R) as a bridge to transplant, those undergoing re-transplant or those who developed intraoperative severe acute hypoxemia (PaO_2_/FiO_2_ ≤150) or hemodynamic instability (mean arterial pressure (MAP) ≤ 60 mmHg and Cardiac Index (CI) ≤ 2.5 L/min/m^2^, despite vasopressors infusion) were considered “high severity” and excluded from data analysis. Patients that intraoperatively maintained respiratory stability (i.e., pH ≥7.25, PaCO_2_ ≤60 mmHg, and PaO_2_/FiO_2_ >150) were considered “low risk” and excluded from this data analysis. Patients that developed intraoperative severe respiratory acidosis with maintained oxygenation function (i.e., pH <7.25, PaCO_2_ >60 mmHg, and PaO_2_/FiO_2_ >150), not associated with hemodynamic instability, were considered at “intermediate” intraoperative severity and their data were selectively analyzed. Data from patients presenting “intermediate” intraoperative severity treated between in the intervals 2007–2010 or 2011–2014 were analyzed as a historical comparison of case-matched cohort. Patients with “intermediate” intraoperative severity treated in the 2007–2010 interval received “standard intraoperative treatment” as compared with those treated in the 2011–2014 interval that received “standard intraoperative treatment + ECCO_2_R.”

For both groups, “standard intraoperative treatment” included protective mechanical ventilation, permissive hypercapnia ≤60 mmHg, and inhaled nitric oxide or a continuous infusion of prostaglandin. Pressure controlled mechanical ventilation (Zeus® Infinity® Dräger, Germany) was set in order to achieve a tidal volume (TV) of 6–8 ml/kg, a plateau pressure <30 cmH_2_O, pulmonary end-expiratory pressure (PEEP) 6–8 cmH_2_O, and FiO_2_ up to obtain peripheral oxygen saturation (SpO_2_) >90% [15–17].

Dobutamine and norepinephrine were titrated to maintain a CI ≥2.5 L/min/m^2^ and a MAP ≥60mmHg. To reduce pulmonary hypertension and to avoid lung ischemic-reperfusion damages, inhaled nitric oxide (iNO) 10–20 ppm was administered (Optikinox® Air Liquide, France) along with prostaglandin E2 continuous intravenous administration at 10–20 ng/kg/min rate [18]. Anemia was treated with red blood cell pack transfusion, for hemoglobin values <9 g/dl. Patients who developed severe intraoperative hypoxemia (PaO_2_/FiO_2_ ≤150) or persistent severe respiratory acidosis (i.e., pH <7.25, PaCO_2_ >60 mmHg) received intraoperative ECMO. When ECMO was initiated during surgery, venous-arterial RotaFlow® (Maquet, Hirrlingen, Germany) was used after femoral artery and vein cannulation: the vein cannula was inserted using percutaneous Seldinger technique and the arterial with surgical preparation. Extracorporeal blood flow was started at 30% of CI. It was then modified according to hemodynamic parameters.

In patients undergoing LT during the 2011–2014 period and presenting “intermediate” clinical severity (i.e., pH <7.25, PaCO_2_ >60 mmHg, and PaO_2_/FiO_2_ >150), CO_2_ removal was achieved using ECCO_2_R (Prolung® device, Estor). The femoral or jugular vein was accessed via a double lumen catheter (14 F; Arrow International Inc. Reading PA) inserted and connected to the extracorporeal circuit. Blood flow was driven through the circuit by a non-occlusive low flow roller pump (80–350 ml/min) through a polimethylpentene oxygenator cartridge membrane connected to an 8 L/min sweep gas flow source delivering FiO_2_ 1.0 oxygen.

The ECCO2R treatment was started at the intraoperative development of severe respiratory acidosis with maintained oxygenation function (i.e., pH <7.25, PaCO_2_ >60 mmHg, and PaO_2_/FiO_2_ >150) that always occurred after the beginning of the first OLV and before the clamping of the pulmonary artery.

Heparin continuous infusion administration of 10–15 IU/kg/h was used as an anticoagulant to maintain activated clotting time (ACT) between 120 and 150s in patients undergoing ECCO2R, while in patients undergoing ECO our anticoagulation target was an ACT between 180 and 200s with an activated partial thromboplastin Time (aPTT) ratio >2.

The following variables were recorded in all patients: MAP, heart rate (HR), mPAP, CI (Vigilance®, Edwards Lifescienses System), central venous pressure (CVP), mixed O_2_ venous saturation (SvO_2_), CO_2_ end-tidal (EtCO_2_), SpO_2_, body temperature, and diuresis. The primary endpoint was the emergency ECMO requirement in the 2 groups. Secondary endpoints were efficacy of ECCO_2_R measured as changes in blood gas analysis (BGA) and impact on systemic and pulmonary hemodynamic (i.e., MAP, HR, and mPAP) recorded every 20 min after the beginning of CO_2_ removal and throughout the intraoperative period. Length of intensive care unit (ICU) stay, duration of postoperative mechanical ventilation, and mortality at 30 postoperative days were also recorded. Complications related to ECCO_2_R use were also recorded, including mechanical complications due to circuit components or pump malfunction and patient-related complications: vascular damages, bleeding, hemodynamic instability, myocardial dysfunction, or cardiac arrhythmias and intravascular embolism.

Continuous variables were described by medians (interquartile ranges) or mean (standard deviation) as appropriate. SPSS Software (IBM) was used for statistical analysis. To evaluate differences between the 2 groups, Fisher’s exact test and *χ*^2^ test with 95% confidence intervals for categorical variables was used; Student’s *t* test was used to analyze continuous variables. Statistical significance was set at a p value lower than 0.05 for all variables. Assuming that 20% of the subjects in the reference population have the factor of interest, and after applying continuity correction, the study would require a sample size of 15 for each group (i.e., a total sample size of 30, assuming equal group sizes), to achieve a power of 80% for detecting a difference in proportions of 0.6 between the two groups (test - reference group) at a two-sided p value of 0.05.

## Results

**Fig. 1:**
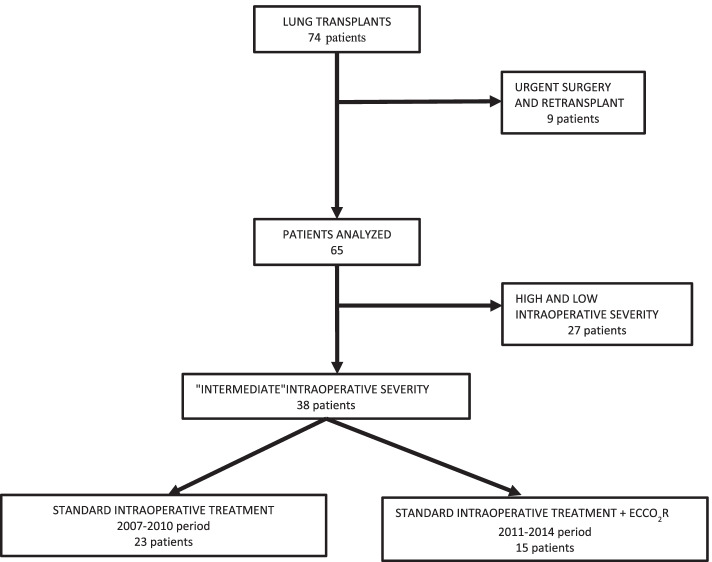
Flow-chart of the study protocol. Legend: Between November 2007 and March 2014, 74 patients underwent lung transplantation, and 9 of these were excluded from the study because of urgent surgery or retransplant. Out of the remaining 65 patients, 38 presented “intermediate” intraoperative severity: 23 patients in the 2007–2010 who “received standard intraoperative treatment,” 15 patients in the 2011–2014 who received “standard intraoperative treatment + extracorporeal carbon dioxide removal (ECCO_2_R). “Intermediate” intraoperative severity: patients who developed intraoperative severe respiratory acidosis with maintained oxygenation function (i.e., pH <7.25, paCO_2_ >60mmHg PaO_2_/FiO_2_ >150), not associated with hemodynamic instability

Seventy-four patients underwent LT at the Transplant Centre of Policlinico Umberto I “Sapienza” University of Rome between November 2007 and March 2014, 9 of these were excluded from the study, 8 received ECMO or ECCO_2_R as a “bridge” to transplant, and 1 was recipient of re-transplant (Fig. 1). Out of the remaining 65 patients, 38 presented “intermediate” intraoperative severity, 23 in the 2007–2010 and received “standard intraoperative treatment,” and 15 in the 2011–2014 and received “standard intraoperative treatment + ECCO_2_R”. There were no significant differences in demographic, baseline hemodynamic, and blood gasses data (Table 1).

**Table 1:** Demographic, clinical characteristics, and variables at baseline

	Standard treatment + ECCO_**2**_R 15 patients	Standard treatment 23 patients	***P***-value
Male/female	8/7	12/11	1.0
Age (years)	43 ± 16	38 ± 12	0.4
Weight (kg)	57 ± 11	59 ± 13	0.61
SLT/DSLT	5/10	5/18	0.47
SLT	Total 5 patients: 3 Pulmonary emphysema 2 Pulmonary fibrosis	Total 5 patients: 1 Pulmonary emphysema 4 Pulmonary fibrosis	
DSLT	Total 10 patients: 9 Cystic fibrosis 1 Pulmonary fibrosis	Total 18 patients: 13 Cystic fibrosis 2 Pulmonary fibrosis 1 Pulmonary hypertension 1 Pulmonary emphysema 1 Bronchiectasis	
pH	7.29 ± 0.08	7.28 ± 0.05	0.73
PaO_2_/FiO_2_	342 ± 151	383 ± 127	0.45
PaCO_2_ (mmHg)	67 ± 23	68 ± 15	0.87
SpO_2_ (%)	98 ± 3	99 ± 1	0.28
BE (mmol/L)	4.3 ± 10.3	3.9 ± 4.1	0.88
HCO_3_- (mmol/L)	29.5 ± 10.2	28.2 ± 3.1	0.65
Lac (mmol/L)	0.7 ± 0.3	0.7 ± 0.3	0.81
HR (beats per minute)	91 ± 13	90 ± 15	0.84
mAP (mmHg)	79 ± 9	74 ± 10	0.14
mPAP (mmHg)	35 ± 12	31 ± 8	0.30
CVP (mmHg)	13 ± 6	12 ± 6	0.91

Values are expressed as mean ± SD

*SLT* single-lung transplant, *DSLT* double sequential lung transplant, *BE* base excess, *Lac* serum lactates, *HR* heart rate, *mAP* mean arterial pressure, *mPAP* mean pulmonary artery pressure, *CVP* central venous pressure

The need for ECMO, in patients that presented “intermediate” intraoperative severity, was more frequent among patients treated in the 2007–2010 period that received “standard intraoperative treatment” alone than in those treated in the 2011–2014 period that received “standard intraoperative treatment + ECCO_2_R” (17/23 vs. 3/15; *p* = 0.004) (Table 2).

**Table 2:** Outcomes

	**Standard treatment plus ECCO**_**2**_**R (*N*** **= 15)**	**Standard treatment (*N*** **= 23)**	***p*-value**	**Odds ratio (95% CI)**
Patients undergoing ECMO, *n* (%)	3 (20%)	17 (73.9%)	0.004	11.3 (2.36–54.5)
Mortality, *n* (%)	3 (20%)	9 (39.1%)	0.08	2.57 (0.56–11.7)
ECMO pts mortality, *n*/*N* (%)	2/3 (66.7 %)	9/17 (52.9%)	0.1	0.56 (0.04–7.44)
	**Standard treatment plus ECCO**_**2**_**R (*N*** **= 12)**	**Standard treatment (*N*** **= 14)**	***p*-value**	
Mechanical ventilation days (day±sd)	3.8±4.2	3.7±2.8	0.33	
ICU length of stay (day±sd)	16.3±15.5	20.6±16.5	0.54	
	**Standard treatment plus ECCO**_**2**_**R (*N*** **= 15)**	**Standard treatment (*N*** **= 23)**	***p*-value**	**Odds ratio (95% CI)**
Tubes damages, *n* (%)	2 (13.3%)	0 (0%)	0.174	8.7 (0.39–195.01)
Surgical revision, *n*/*N* (%) (ECMO bleeding)	1/3 (33.3%)	2/17 (11.77%)	0.36	0.75 (0.22–62.77)

In the upper side of the box, the rate of ECMO treatment in the standard treatment and standard treatment plus ECCO_2_R groups; in the middle side of the box, the mechanical ventilation days and postoperative ICU length of stay in the 2 groups (patients who had ECMO treatment were excluded); in the lower side of the box, the complications occurred in the 2 groups

Urgent ECMO was used in 20 patients, 3 (20%) in standard intraoperative treatment + ECCO_2_R group, and 17 (73.9%) in standard intraoperative treatment group (p: 0.004 OR = 11.3 CI = 2.36–54.5) (Table 2).

In the “Standard intraoperative treatment” group, acidosis and hemodynamic instability were determining factors for urgent ECMO: in 8 patients, it happened during the first OLV period, in 4 patients during the first pulmonary artery clamping, in 2 patients during the second OLV time, in the last 3 patients after the second pulmonary artery clamping.

In the “Standard intraoperative treatment + ECCO_2_R” group, causing factors for ECMO were mainly respiratory adverse events, during the second pulmonary artery clamping, related to the PGD of the first lung implanted.

**Fig. 2:**
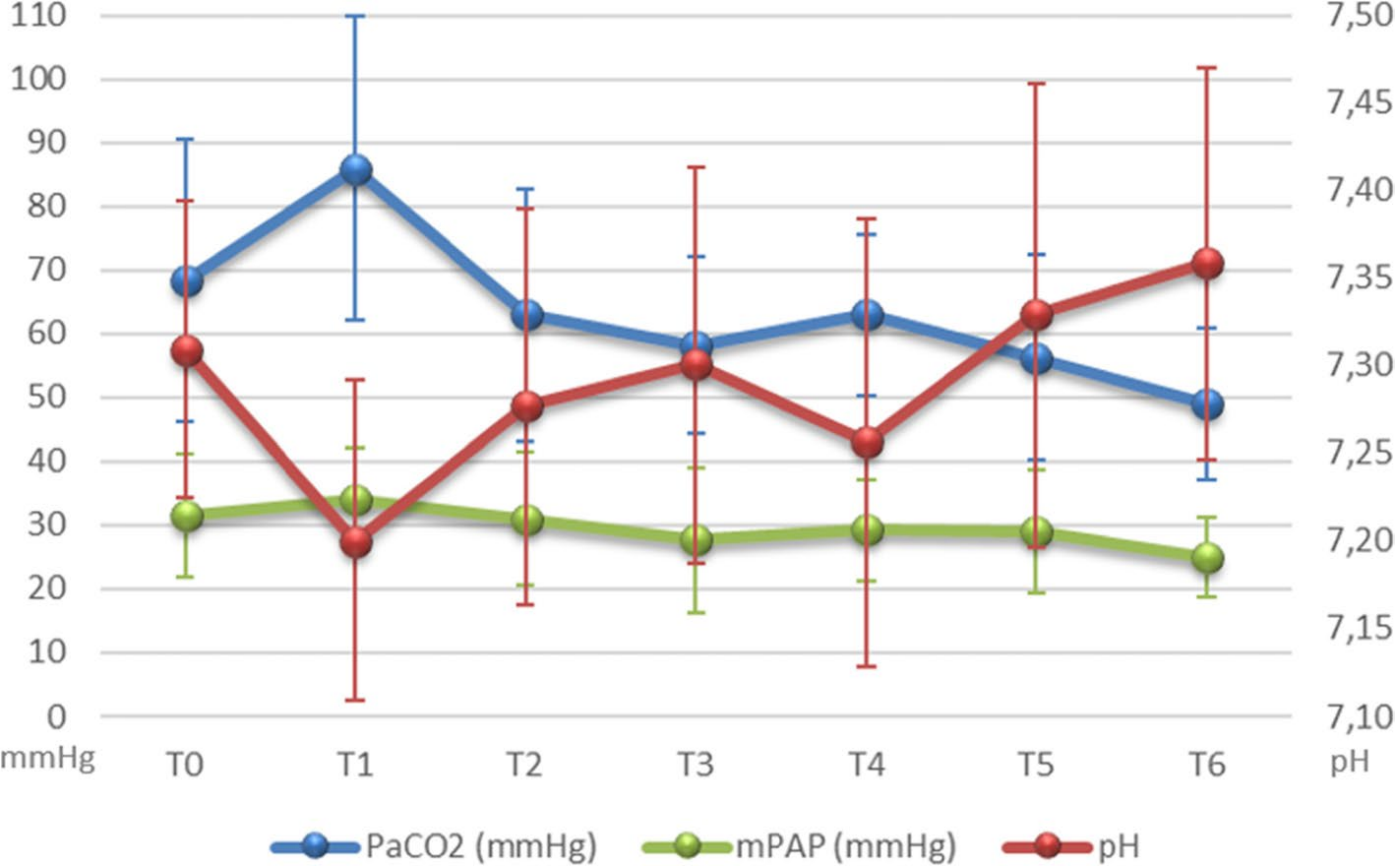
Changes in PaCO2, mPAP and pH in the 15 patients undergoing ECCO2R. Legend: PaCO2 = Arterial Carbon Dioxide Partial Pressure; mPAP = mean pulmonary artery blood pressure; T0 = double lung ventilation at the beginning of LT; T1 = single lung ventilation (preclamping of the pulmonary artery); T2 = clamping of the pulmonary artery; T3= after 40 min from clamping; T4 = after 90 min from clamping; T5 = after 120 min from clamping; T6 = declamping of the pulmonary artery; primary axis (on the left) = values of PaCO2 and mPAP in mmHg; secondary axis (on the right) = values of pH. All values are reported as mean and standard deviation. ECCO2R was started between T1 and T2 and continued until the end of the surgery

Data analysis of hemodynamic and respiratory variables showed a decrease in mPAP and PaCO_2_ values along with an improvement in pH values, starting after 20 min and thereafter throughout the intraoperative period, while systemic hemodynamic variables and PaO_2_/FiO_2_ remained stable (Table 3, Fig. 2). The 30-day mortality rate was 20% (3/15) in the “standard intraoperative treatment + ECCO_2_R” group and 39% (9/23) in the “standard intraoperative treatment” (*p* = 0.55). Mortality among patients who received ECMO was similar among “standard intraoperative treatment + ECCO_2_R” and “standard intraoperative treatment” (3/15, 20% vs 9/23, 39.1%).

**Table 3:** Cardiorespiratory variables in the 15 patients that received ECCO2R

	T0	T1	T2	T3	T4	T5	T6
**HR** (beats per minutes)	87 ± 15	95 ± 21	94 ± 20	98 ± 19	104 ± 19	105 ± 19	104 ± 17
**MAP** (mmHg)	77 ± 10	76 ± 11	71 ± 11	72 ± 11	73 ± 11	73 ± 12	76 ± 9
**mPAP** (mmHg)	31 ± 10	34 ± 8	31 ± 10	28 ± 11	29 ± 8	29 ± 10	25 ± 6
**PVC** (mmHg)	12 ± 5.3	13 ± 4	13 ± 5.5	12 ± 5.4	13 ± 5.4	14 ± 5.9	14 ± 5
**CI** (l/min/m^2^)	2.7 ± 0.8	2.9 ± 1	3.0 ± 0.9	3.1 ± 1	3.6 ± 1	3.1 ± 1.2	3.0 ± 0.7
**SvO2** (%)	82 ± 8	79 ± 11	82 ± 9	83 ± 6	82 ± 7	79 ± 10	81 ± 7
**pH**	7.31 ± 0.1	7.20 ± 0.1	7.28 ± 0.1*	7.30 ± 0.1	7.26 ± 0.1	7.33 ± 0.1	7.36 ± 0.1
**PaO**_**2**_**/FiO**_**2**_	359 ± 131	238 ± 179	309 ± 169	340 ± 171	155 ± 90	210 ± 167	264 ± 164
**PaCO**_**2**_ (mmHg)	68 ± 22	86 ± 24	63 ± 20*	58 ± 14	63 ± 13	56 ± 16	49 ± 12
**SpO**_**2**_ (%)	99 ± 2	97 ± 3	99 ± 2	99 ± 4	93 ± 11	97 ± 7	99 ± 1
**BE** (mmol/l)	6.1 ± 9	2.8 ± 3.2	1.9 ± 9	1.0 ± 6	0.7 ± 7	2.1 ± 6	1.2 ± 5
**HCO3**^**-**^ (mmol/l)	31 ± 8	28.7 ± 3.7	27 ± 8	26 ± 4	26 ± 6	27 ± 5	26 ± 4
**Lac** (mmol/l)	0.8 ± 0.5	0.6 ± 0.3	1.2 ± 0.7	1.7 ± 1.2	5.1 ± 13	2.5 ± 2.1	4.7 ± 3.9

Cardiorespiratory parameters in different phases of LT

ECCO2R was started between T1 and T2

*HR* heart rate, *MAP* mean arterial pressure, *mPAP* mean pulmonary artery blood pressure, *PVC* central venous pressure, *CI* cardiac index, *SvO*_*2*_ mixed venous oxygen saturation, *BE* bases excess, *Lac* lactates, *T0* double lung ventilation at the beginning of LT, *T1* single lung ventilation (preclamping of the pulmonary artery), *T2* clamping of the pulmonary artery, *T3* after 40 min from clamping, *T4* after 90 min from clamping, *T5* after 120 min from clamping, *T6* declamping of the pulmonary artery

*p< 0.05 VS single lung ventilation

Complications of ECCO_2_R included tube damages occurred twice with the system, but never with ECMO circuit. Three patients required wound and thoracic surgical revision because of ECMO-related bleeding (1 in the “standard intraoperative treatment + ECCO_2_R” group and 2 in the “standard intraoperative treatment” group). No other complications were reported.

## Discussion

This case-matched cohort retrospective study, in patients undergoing LT and presenting “intermediate” clinical severity, originally reports that intraoperative ECCO_2_R decreases emergency ECMO requirement. In these patients, when intraoperative respiratory acidosis develops, ECCO_2_R use safely and effectively blunts pH/PaCO_2_ changes and associates with reduced mPAP (Fig. 2). These results confirm the efficacy of ECCO_2_R in CO_2_ removal, already proven in ultraprotective ventilation in chronic obstructive pulmonary disease (COPD) and in ARDS patients, and extend its role to the perioperative treatment of patients undergoing LT [19–21]. Limited and promising evidence is currently available on the perioperative use of ECCO_2_R in thoracic surgery, and these include a 69-year-old man undergone left pneumonectomy treated with resection of a single right upper lobe lesion; and the report of ECCO_2_R adjunct to conventional treatment as a bridge to LT [22]. The ECCO_2_R use has also been tested in the immediate postoperative period in case PGD when conventional therapies (ventilatory support, inhaled nitric oxide administration, and intravenous prostaglandin) were not sufficient to provide adequate gas exchange and control pulmonary hypertension [23–25].

Patients selected for this cohort study represent an “intermediate group” in terms of clinical severity, do not present severe hypoxemia, are not receiving preoperative extracorporeal lung support with either ECMO or ECCO_2_R, and are not scheduled for re-transplant neither present with severe refractory arterial hypotension or reduced CI; on the other hand, the respiratory function was severely compromised and associated with advanced respiratory acidosis. For this reason, we consider that the presented data intended to fulfill the need for a therapeutic intraoperative “precision medicine” strategy [26]. In both groups, a protective mechanical ventilation strategy was set according to the recommendations to minimize the ventilator-induced lung injury and in particular hyperinflation [17]. Prior to inclusion mechanically ventilated patients presented a hypercapnia (PaCO_2_ >60mmHg) and respiratory acidosis (pH< 7.25), while ventilation was optimized within the limitations imposed by lung-protective ventilation [17, 27]. Permissive hypercapnia with moderate respiratory acidosis (PaCO_2_ 40–60 mmHg with pH >7.25) was considered acceptable in order to reduce the risk of dynamic hyperinflation, barotrauma, and volutrauma [28]. Patients presenting severe intraoperative respiratory acidosis (i.e., pH <7.25 and PaCO_2_ >60 mmHg) were free from metabolic and perfusion mismatch, maintaining a MAP ≥60 mmHg and CI ≥2.5 l/min/m^2^, with or without vasopressors infusion [27, 28]. Patients with severe hypoxemia PO_2_/FiO_2_ <150 were excluded and underwent to an ECMO support.

### Study limitations

The present study has some limitations: first, it is a single-center case-matched cohort retrospective study. This methodological approach does not provide evidence that possesses an equivalent strength than those derived by a randomized controlled trial; furthermore, we could not apply matched analysis techniques with propensity score without significantly reducing the sample size. Nevertheless, the number of recruited patients (based on a dedicated sample size calculation) is sufficient to support the presented results and it should be considered as a preliminary experience for designing a multicenter prospective study.

## Conclusion

In conclusion, intraoperative ECCO_2_R use in patients undergoing LT is safe and along with “optimal” mechanical ventilation and hemodynamic management, effectively contributes to prevent respiratory acidosis and hypercapnia. This approach, when accomplished in patients presenting with “intermediate severity” and compared to ECMO has the advantage of using smaller intravascular cannulation and lower blood flow, thus exposing to a reduced risk for vascular damage and bleeding complications. The safety and efficacy of intraoperative ECCO_2_R use potentially make this treatment a safe and useful step into an escalating step-wise work-up to support respiratory function in patients undergoing LT. Future studies are necessary to define the clinical indications, to improve the design of membrane CO_2_ removal, and to further reduce invasiveness.

## Data Availability

The datasets used and/or analyzed during the current study are available from the corresponding author on reasonable request.

## Abbreviations

ECCO_2_R: Extracorporeal carbon dioxide removal
ECMO: Extracorporeal membrane oxygenation
CO_2_: Carbon dioxide
LT: Lung transplantation
PaCO_2_: Arterial carbon dioxide partial pressure
mPAP: Mean pulmonary artery pressure
CPB: Cardiopulmonary bypass
PGD: Primary graft dysfunction
ARDS: Acute respiratory distress syndrome
TV: Tidal volume
PEEP: Pulmonary end-expiratory pressure
FiO_2_: Fraction of inspired oxygen
SpO_2_: Peripheral oxygen saturation
MAP: Mean arterial pressure
CI: Cardiac Index
iNO: Inhaled nitric oxide
ACT: Activated clotting time
aPTT: Activated partial thromboplastin time
HR: Heart rate
CVP: Central venous pressure
SvO_2_: Mixed oxygen venous saturation
EtCO_2_: End-tidal CO_2_
BGA: Blood gas analysis
ICU: Intensive care unit
COPD: Chronic obstructive pulmonary disease
OLV: One lung ventilation
SLT: Single lung transplant
DSLT: Double sequential lung transplant
